# Chest Drainage Therapy: What Comes out of Pandora’s Box Can Affect Patient Outcomes

**DOI:** 10.3390/jcm11185311

**Published:** 2022-09-09

**Authors:** Alberto Antonicelli, Fabrizio Monaco, Angelo Carretta, Bryan M. Burt, Joshua R. Sonett, Giulia Veronesi

**Affiliations:** 1 Thoracic Surgery Department, IRCCS San Raffaele Hospital, 20132 Milan, Italy; 2 The National Coalition of Independent Scholars (NCIS), Brattleboro, VT 05301, USA; 3 Cardiothoracic Intensive Care Unit, IRCCS San Raffaele Hospital, 20132 Milan, Italy; 4 Division of Thoracic Surgery, Baylor College of Medicine, Houston, TX 77030, USA; 5 Section of General Thoracic Surgery, Columbia University Irving Medical Center, New York, NY 10032, USA

**Keywords:** chest drainage unit, intrapleural pressure, air leaks, complication, target therapy

## Abstract

**Background:** Over the last 100 years, the original three-bottle chest drainage system has been variously engineered into compact disposables and electronic units. Clinicians are now surrounded by a plethora of different types of systems, but little is known about the way that they work and perform. Thus, we sought to test the performance of the most commonly used chest drainage units under conditions that are relevant to clinical practice. **Methods:** A pleural space environment simulator was built. Thirty-two units were tested under four clinical scenarios: air leak interpretation during quiet breathing and after obstructed inspiration (−5 to −150 cmH_2_O), a buildup of negative pressure (−100 cmH_2_O), a bronchopleural fistula (10 L/min) and the need for effective external suction in the presence of air leakage. Twenty-five units were “traditional” thoracic drainages, five were “digital” low-flow/low-vacuum pumps and two were hybrids (a combination of the two). According to the design of the seal and of the suction control, the units were classified as wet-wet, wet-dry and dry-dry. **Results:** All wet units showed reverse air flow, with the potential to mimic an air leak when there was none. Ten wet units showed no automatic negative pressure relief features, while five dry-dry did but were slow to react. Ten wet and five dry-dry units showed no capability to handle a 10 L/min leak, as they were restrictive to flow (peak pressure up to 55 cmH_2_O). Only seven dry-suction units were able to maintain the set suction at high airflow rates (>20 L/min). **Conclusions:** Different chest drainage unit designs lead to different performances, some of which may negatively impact patient outcomes. This sounds the call to tailor our clinical practice for the individual patient. A paradigm shift to better understand all components of pleural physiology post-surgical intervention on this relatively neglected topic is needed to improve our daily practice.

## 1. Introduction

Adequate drainage of the pleural space is the cornerstone of good post-operative management [1]. Several types of chest drainage systems are commercially available but systematic investigation of how they work is relatively sparse [2,3,4].

Chest drainage units (CDUs) that rely on water to make a seal and therefore are called wet-systems, or simply underwater sealed drains (UWSD), are very common. Although they exist in various shapes, all of them are based upon the Bülau-principle. The Bülau-principle is a therapeutic drain using a permanent siphon generated by a Heber-drain within a closed system. The Heber-drain works according to the Heber-principle using hydrostatic pressure [5]: the vertical height between the chest and the CDU (hydrostatic column) determines the level of sub-atmospheric pressure applied to the patient.

CDUs that do not rely on water to make a seal are called dry systems. In theory, the shift from “wet” to “dry” seal technology was intended to introduce a standardized framework to support patient recovery. In reality, dry seals have an entirely different structure and mechanism of function. Miniaturized Heimlich and electronic valves are indeed variously integrated in ‘analogue’ and ‘digital’ CDUs, respectively. This undesirable variability is an example of noise, the ubiquitous and often-ignored burden in clinical decision-making.

The choice of a medical device requires evidence which can be scarce and not fully available [6,7]. In addition, a “one size fits all” CDU does not yet exist and therefore it is reasonable that one design can be adequate for one patient and not for another. Thus, it is important for clinicians to be aware of the pros and cons of the most common CDUs available on the market to improve patient outcomes [8,9].

Clinicians shall expect the following basic characteristics from the CDUs they use: to be designed to facilitate correct interpretation of changes in clinical status; to provide pressure protection, quickly and automatically; and to evacuate the highest airflow at the lowest pressure (low resistance).

This review is focused on two aspects: (1) rational choosing of the model of CDU among those commercially available and (2) understanding the use of all models along with data interpretation. Finally, we analyzed a new device capable of helping in removing barriers to rational choosing and optimal use of CDUs.

## 2. Materials and Methods

Thirty-two CDUs from ten manufacturers were collected. Relevant design characteristics were observed by sawing them apart (Table 1).

CDUs were classified based on their mechanism of function (Table 2): “wet-wet”, where water is used to establish the seal (wet-seal) and to set the amount of wall suction (wet-suction); “wet-dry”, where water is used to establish the seal while a mechanical component is used to set the amount of wall suction (dry-suction); “dry-dry”, where mechanical or electronic components are built-in to create the seal (dry-seal) and to set the amount of wall or independent suction, respectively. UWSD were subclassified depending on the number of chambers as in one-bottle systems the Heber pipe is in direct continuity with the connecting tube (hence water can potentially rise all the way up to the pleural space), in multi-bottle systems the water seal is physically separated from the fluid collection chamber (but water can be siphoned out of the water seal chamber and into the collection chamber) and in compact systems a float valve on top of the water column prevents water from spilling over.

**Figure 1:**
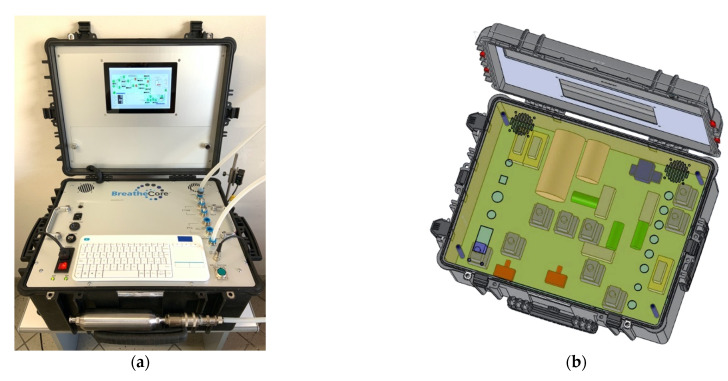

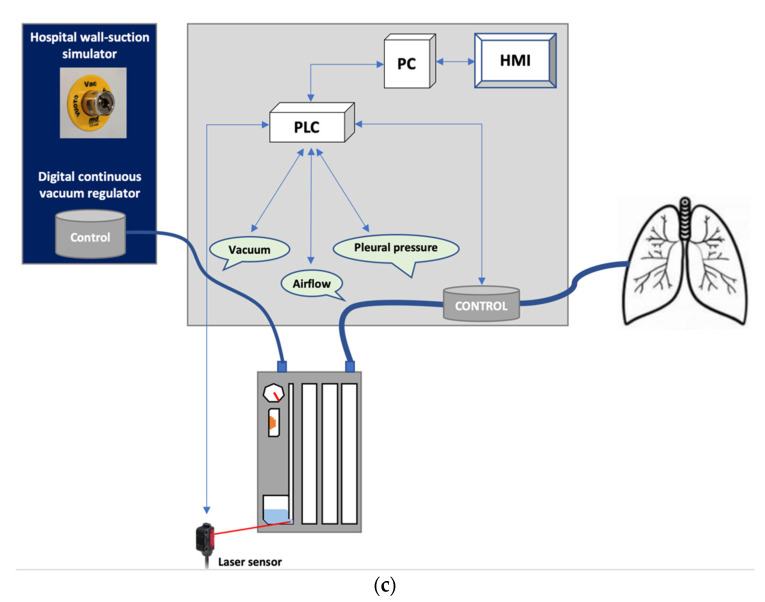
(**a**,**b**) Picture and Computer Aided Design of the BreatheCore^TM^ Gray-box Testing and Simulator System. (**c**) Experimental setup for CDUs testing and bench model for chest drainage. PLC = Programmable Logic Controller, PC = embedded Personal Computer, HMI = Human Machine Interface.

To test the CDUs, a pleural space environment simulator was built (Figure 1a). The experimental setup is illustrated in Figure 1b. A custom motherboard was engineered to assemble a programmable logic controller (PLC) interconnected with two independent pumps (0 to 3 L and 3 to 20 L/min), solenoid electro-valves, alloy-buffer chambers, two flow meters (0.07 and 0.12 psid pressure drop at full scale flow, response time 10 ms) and two pressure sensors (accuracy = 0.2 and 0.04% at full-scale range, respectively). The pneumatic circuit was made by tubing with a minimum internal lumen diameter of 8 mm. Hospital in-wall suction was recreated building a digitally controlled high-flow high-vacuum pump with dedicated flow and pressure sensors. An ad hoc software interface was developed to create various breathing patterns. Normal and pathological conditions observed in the real life with unassisted spontaneous breathing or mechanical ventilation were simulated. A laser sensor was added to detect air bubbles when testing UWSD. BreatheCore^TM^ gray-box testing was chosen for two reasons: (i) because it is a method that can be used to search for the defects, if any, due to improper structure or improper usage of applications and (ii) because our knowledge of how each of the CDUs worked was limited. Reliability was tested through five repetitions (test–retest reliability coefficient >0.9). Proper granularity of the measurements was guaranteed by high-resolution data logging (17 per second). Built-in controls included machine checks to automatically detect errors caused by equipment malfunctioning. Every CDU was tested under three conditions which have clinical implications. (i) Air movements through UWSD were simulated, breathing at 15 acts per minute at an intrapleural pressure from −5 to −150 cmH_2_O. (ii) Negative and positive pressure build up, which can be encountered in the event of obstructed inspiration and/or bronchopleural fistula, was simulated with an air pocket of 0.75 L at −100 cmH_2_O and a free airflow from 3 to 15 L/min, respectively. (iii) External applied suction generation and the ability to maintain this through the CDU even in the presence of air leaks was tested applying a vacuum from −20 to −400 cmH_2_O to the suction outlet of wet-wet and wet-dry thoracic drainages.

## 3. Results

### 3.1. How Air Moves along the Entire UWSD System (Wet-Wet and Wet-Dry CDUs)

The blue water rose and fell in the analogue manometer synchronously with the simulated patient’s breathing, reflecting air moving at different pressures. During inspiration, atmospheric air could backflow becoming visible as bubbles, a situation known as reverse-airflow (RAF). Two mechanisms were observed (Table 3). In five CDU designs (High capacity by Bio-Thorametrix, Aquaseal and Thoraseal III by Covidien, Rome/Venice by Eurosets, Compact/Variant/Simple3/Simple and Simple plus by Redax), RAF occurred during each inspiration by bending of the water surface, despite maintaining water in the reservoir. In all CDUs (except for the Altitude and the Sentinel Seal), backflow of air occurred as a result of complete emptying of the reservoir.

### 3.2. How CDUs Manage the Build-Up of Large Negative Intrapleural Pressure

In a one-bottle UWSD (Single by Bio-Thorametrix, One bottle and Thora-Seal I and II by Covidien, Thoraflow by Meditea, Chest by Redax), water was raised real-time and unrestricted, to the point that it could be sucked into the simulated pleural space at pressures exceeding the vertical length of the Heber pipe plus the connecting tube (~170 cm).

**Figure 2:**
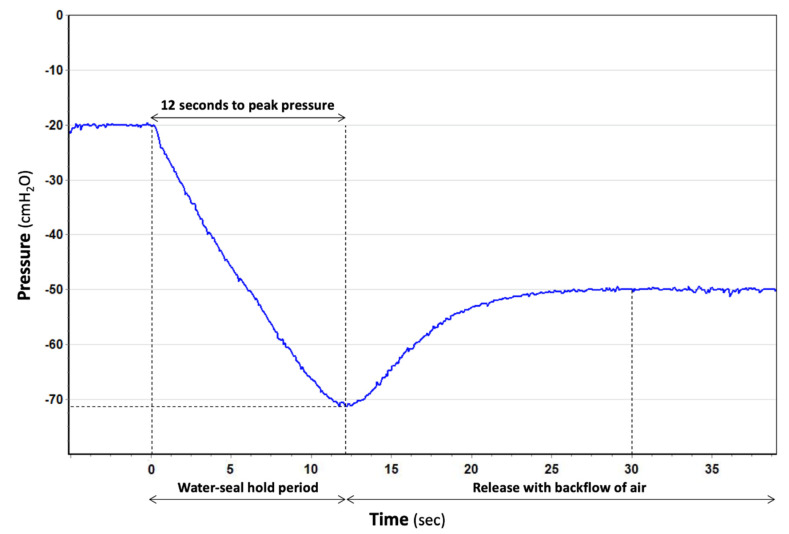
An example of automatic negative pressure protection partially provided by a regulated RAF in a compact UWSD (the Oasis by Atrium-Maquet, blue line). Testing started at −20 cmH_2_O of external suction applied. When −100 cmH_2_O of intrapleural pressure was simulated, the Oasis held the seal for 12 s and then broke it (at −70 cmH_2_O peak), lessening such negativity to a safer −50 cmH_2_O.

In a compact UWSD (Oasis/Ocean by Atrium-Maquet, High capacity by Bio-thorametrix, Altitude/Sentinel Seal/Aqua-Seal/Thora-Seal III by Covidien, Rome/Venice by Eurosets, Compact/Variant/Simple3/Simple/Simple Plus by Redax, and Pleur-evac A6000 Cactus/A7000 by Teleflex), water was raised real-time and unrestricted for the length of the water column manometer (which varied between 15 and 25 cmH_2_O), on top of which a float valve shut-offed. Float valves were shaped like a cone or a ball. The former would always provide a hermetic seal, preserving the water seal but contributing to the build-up of large intrapleural pressure (Altitude/Sentinel and Aqua-Seal by Covidien). The latter would plug in the circumference of a plastic hole and, only if a notch was present would it still allow water to raise, until emptying of the reservoir with consequent RAF (Oasis/Ocean by Atrium-Maquet, Pleur-evac A6000 Cactus/A7000 by Teleflex) (Figure 2).

The higher the negativity, the shorter the time needed to break the water seal, achieving, therefore, pressure build-up protection. High capacity by Bio-thorametrix, Rome/Venice by Eurosets and Thora-Seal III by Covidien allowed RAF at relatively low levels of intrapleural pressure (24 to 28 cmH_2_O) maintained for an average of 35 s. Compact/Variant/Simple3/Simple/Simple Plus by Redax allowed RAF at a much higher level of intrapleural pressure (131 cmH_2_O) maintained for 244 s.

**Figure 3:**
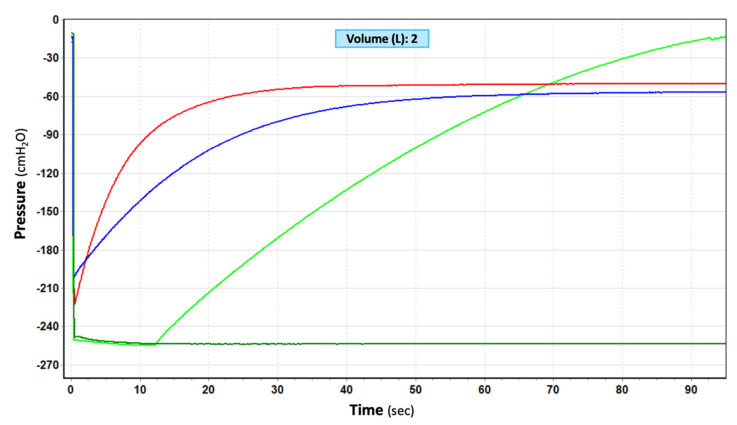
Mechanisms of function and time needed to lower a build-up of −250 cmH_2_O in three different CDUs. Two dry-dry CDUs (Express by Atrium-Maquet, red line; Pleur-evac Sahara 1100 by Teleflex, blue line) and one compact UWSD (Argyle Aquaseal by Covidien/Cardinal Health, green lines) are shown. Aqua-seal can provide pressure relief only if the valve is manually activated (light vs. dark green lines).

**Figure 4:**
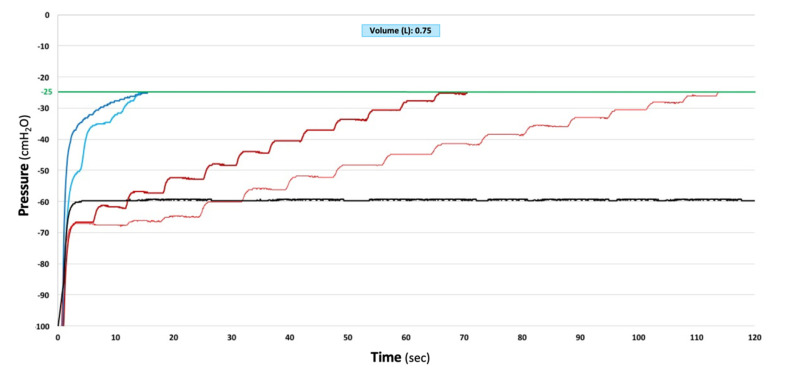
Patterns and time needed to reestablish physiologic intrapleural pressures values (−20 +/− 5 cmH_2_O, green line) in five different CDUs simulating a build-up of −100 cmH_2_O. One hybrid CDU (Drentech EVO by Redax, black line) and four electronic systems (Thopaz and Thopaz+ by Medela, light and dark red lines; C051 and S201 by ATMOS, light and dark blue lines) are shown.

**Figure 5:**
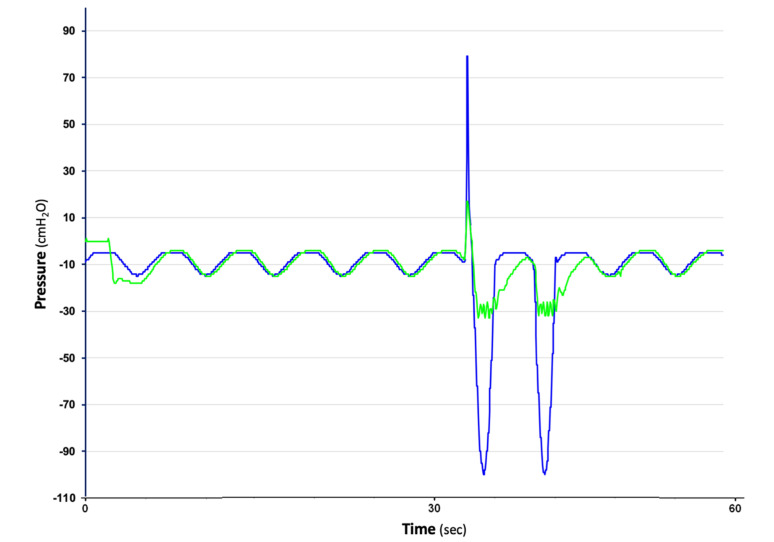
Quite breathing (−5 to −15 cmH_2_O), coughing (300cmH_2_O) and obstructed breathing (−100 cmH_2_O) was simulated. The resulting intra-pleural pressure status is shown (blue line), with a hybrid CDU (Drentech EVO by Redax) being tested. Real-time positive and negative pressure protection (green line) was provided by electronically controlled, high-flow low-resistance, valves incorporated in the BreatheCore^TM^ System.

In those CDUs where the seal was made by a rubber flutter one-way valve or by an electronic vacuum pump (dry-dry), two automatic different vacuum release technologies were observed. In the former, an analogue pressure relief valve remained closed until −70 cmH_2_O, when it cracked open to the atmosphere and remained, so allowing flow as over pressure increased; the valve closed back again at −50 cmH_2_O, showing an operating hysteresis of 20 cmH_2_O. In the latter, a sensor had to detect the negativity first. Then, software had to operate an electronic pressure relief valve to cyclically open and close until system pressure was reduced to the set pressure. Dry-dry CDUs marked as thoracic drainage did not provide any pressure relief until −70 cmH_2_O, beyond which they were quick to react, providing continued pressure relief with a relatively low restriction to flow. The Express by Atrium-Maquet was quicker than the Pleur-evac Sahara 1100 by Teleflex (Figure 3). Dry-dry CDUs marked as low-flow/low-vacuum pumps provide pressure relief at any value beyond the set pressure, but are slow to react and highly restrictive to flow (Figure 4). Proper changes to CDU designs led to instant pressure protection, lowering an initial −100 cmH_2_O to a safer −30 cmH_2_O in 0.5 s (Figure 5).

### 3.3. How CDUs Manage the Accumulation of Positive Intrapleural Pressure

**Figure 6:**
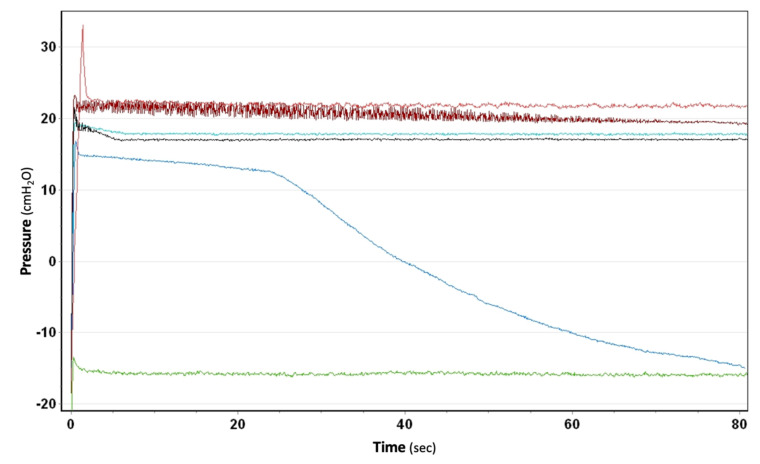
Peak-pressures and time needed to provide positive-pressure protection in five different CDUs simulating a broncho-pleural fistula with a free airflow rate of 10 L/min. One compact UWSD (Oasis by Atrium-Maquet, green line), one hybrid (Drentech EVO by Redax, black line) and four electronic CDUs (Thopaz and Thopaz+ by Medela, light and dark red lines; C051 and S201 by ATMOS, light and dark blue lines) are shown.

With a free airflow rate of 10 L/min at an external applied suction set at −20 cmH_2_O, CDUs classified as thoracic drainage developed a peak-pressure ranging from 5 to 8 cmH_2_O while ‘hybrids’ and those classified as low-flow/low pressure pumps developed a peak-pressure ranging from 35 to 55 cmH_2_O (Figure 6). All electronic CDUs allowed the normally negative pressure intrapleural space to instead remain positive at length (>40 sec). The only electronic CDU capable to evacuate a 10/min leak was the S201 by ATMOS.

### 3.4. How Suction Is Generated and Whether the Set Amount Is Effectively Maintained through the Canister, Even in the Presence of Air Leaks

In those CDUs where the suction control was made by water (wet-wet), free air flowrates of 15 L/min were evacuated with the water level set at −10 cmH_2_O with −100 cmH_2_O of wall suction. A higher flowrate e.g., 25 L/min, and the ability to set higher vacuum, e.g., −20 cmH_2_O, was possible. Wall suction had to be titrated so that the fluid in the suction chamber would bubble gently. Given the intrinsic nature of bubbling, the generated suction was rapidly intermittent (undulated line). More work and time were needed to adjust the water level in the suction control chamber, and the CDU was noisier. The higher the wall suction, the sharper the undulations, with a higher probability of the water back-flowing. In addition, the water level could drop due to over vigorous bubbling requiring topping up of the fluid level.

In those CDUs where the suction control was made by a dial control knob (wet-dry and dry-dry), free air flowrates of 15 L/min were evacuated at a set of −10 cmH_2_O with −100 cmH_2_O of wall suction. A higher flowrate, e.g. 25 L/min, and the ability to set higher vacuum, e.g. −40 cmH_2_O, was possible with −200 cmH_2_O of wall suction or higher. Given the absence of water in the third chamber, the generated suction was steady and precise (flat line). When wall suction was adequate for the set vacuum, a suction indicator in the form of an expanding bellow, a floating cylinder or the word ‘Yes’ would appear, depending on the CDU model. Less work and time were needed to adjust the desired vacuum in the suction control chamber, and the CDU was quieter. Thanks to a fourth chamber working as a patient assessing manometer, the generated suction was steady and both precise and accurate only in one CDU (Sentinel Seal by Covidien).

In those CDUs where the suction control was made by a vacuum pump (dry-dry), free air flowrates of 4.5 L/min were evacuated at a set of −10 cmH_2_O. A higher flowrate was not possible. The ability to set higher vacuum, e.g. −40 cmH_2_O, was possible but no changes in flowrates were observed. Given the nature of electronic vacuum pumps, the generated suction was rapidly intermittent (undulated line). Less work and time were needed to adjust the desired vacuum on the display, and the CDU was quieter.

## 4. Discussion

The main finding of the present investigation is that in a laboratory setting only three commercially available chest drainage units met clinical expectations: the wet-wet three-bottle system (Bülau), and two wet-dry compact units, the Oasis (by Atrium-Maquet) and the Pleur-Evac A6000 Cactus (by Teleflex).

**Figure 7:**
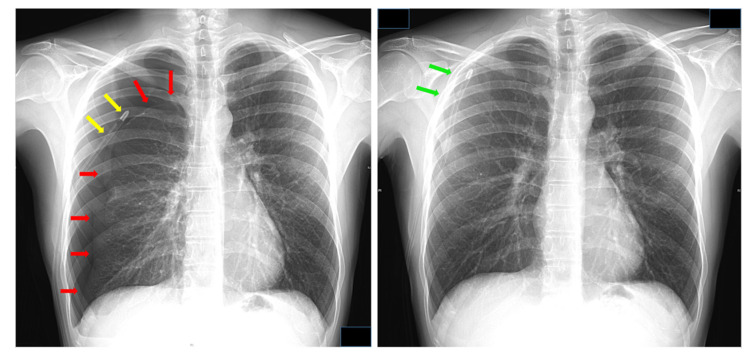
(**left**) Pneumothorax induced by emptying the water contained in the water-seal reservoir in a compact UWSD (Rome by Eurosets). Red arrows: lung surface; yellow arrows: chest tube. (**right**) Pneumothorax resolved by switching to a dry-dry low-flow/low-pressure pump (Thopaz by Medela). Green arrows: lung surface. Data kindly provided by Dr. Alessandro Brunelli.

We believe that the effects of lung resection can be a direct function of the CDU used. For example, excessive build-up of negative intrapleural pressure can occur anytime we have a ‘stiff lung’, such as in patients with lung fibrosis [10] and in the acute phase after lung volume reduction surgery [11], or in the mechanically ventilated patient during and after extubation [12], when air leaks occur and an external source of vacuum is needed, [13] and whenever chest tube stripping is performed [14]. This, altogether, makes it important for a release technology to be built into the CDU we use. Some UWSD systems rely on breaking the water-seal to allow atmospheric air to backflow. Some authors have advised against this mechanism because air can be misinterpreted as persistent air leaks [15], to the point that ways have been developed to distinguish ‘true’ from ‘false’ air leaks [16]. We believe that the reverse air flow can actually be a safety feature, and that it does not set the stage for prolonged hospitalization as long as it is automatically regulated (Figure 2). The response time is crucial; indeed, when RAF occurs too early it leads to pneumothorax (Figure 7) whereas a delay in response generates a buildup of negative pressure which is clinically associated with discomfort and lung tears.

This timed emptying mechanism can be achieved with proper design of the water seal chamber in any UWSD. Some dry-seal systems rely on high negative pressure mechanical valves that open automatically around −70 cmH_2_O, preventing therefore any further increase in negative pressure. Either way, the ideal seal should be able to react real-time to changes in pressure and to provide high-flow low-resistance pathways for air to take [17]. The same considerations apply to positive pressure relief valves, especially in those CDUs that are highly restrictive to flow [3] and slow to react (“digital” low-flow/low-vacuum pumps and “hybrids”).

Another aspect that influences air flowrate is the lumen of the entire tubing. Since flow has a relationship to the fifth power of the radius of the drainage tube, a tube with 6 mm internal diameter is the minimum required to allow a maximum flow of 15.1 L/min of air at an applied pressure of −10 cmH_2_O [18]. Despite this, plastic connectors with a 4mm internal diameter are still used between the chest drain and the connecting tube. Furthermore, according to ISO [7], dry-dry CDUs known as “digital drains” are classified as ‘low-flow/low-pressure pumps’, hence they are not required to guarantee more than 5 L/min of airflow (which is, instead, a requirement for those CDUs classified as ‘thoracic drainages’). Although they came to the market as a better alternative to traditional plastic disposables, they are accepted for use in all patients and perform at standards that are far lower than those of traditional thoracic drainages. As thoracic surgeons, regardless of how CDUs are named and advertised by manufacturers or classified by authorities, we ought to use thoracic drainages as uniformly as possible to ensure patient safety.

Real-time visual feedback on air-leaks and pleural pressure swings is another important feature that guides clinical decision-making. A reservoir filled with water is currently a requirement for achieving this, and UWSD systems set an example as described earlier. In some dry-dry systems, a reservoir is built-in for water to be added to allow air leak visualization as bubbles. This applies to the Express (by Atrium/Maquet), to the Sahara 1100 (Pleur-evac line by Teleflex) and to the S201 (by ATMOS). The first two ones are thoracic drainages with a rubber flutter one-way valve built-in in order to make the seal. The latter is a low-flow low-vacuum pump with a detachable canister that incorporates a water reservoir for temporary gravity drainage. Of note, in all three CDUs water oscillations in the analogue manometer do not reflect pleural pressure swings due to the intrinsic design of the seals. The only dry-dry CDU that provided a digital real-time visual feedback on pleural pressure swings was the DigiVent thoracic drainage (by Millicore, Sweden) [19,20]. The DigiVent was also capable of distinguishing an active air leak from a pleural space effect [21]. Unfortunately, it is no longer on the market. DigiVent technology was acquired by Medela (Baar, Switzerland) but neither of their CDUs (Thopaz and Thopaz+) offer this feature. Clinicians are therefore blind to the intrapleural pressure status of their patients, as data can only be downloaded on a PC interrupting chest drainage therapy or after chest drain removal.

Effective pleural drainage also depends on the pressure gradient between the pleural space and the CDU. The hydrostatic column in the connecting tube can generate as much sub-atmospheric pressure as the vertical distance between the chest and the CDU. The pressure gradient can be increased to enhance drainage by lowering the level of the CDU below the patient and by adding a source of vacuum. The latter overcomes the detrimental effect of air pockets produced by dependent loops which can break the continuity of the liquid column, causing the loss of sub-atmospheric pressure and thus impeding the flow of air [22,23,24]. There are two sources of vacuum: hospital in-wall outlets and portable units. The first relies on large industrial-scale vacuum pumps generating ‘wall’ suction in each hospital room (around −500 cmH_2_O). This pressure is far greater than that required for thoracic drainage, so pressure regulators are mounted to the wall. Ultimately, each CDU has ways to further regulate suction on demand, either with a column of water or by dialing a control knob in the suction control chamber. The second can consist either of add-ons like the PALM-EVO by Redax and the PSU by Rocket, or vacuum pumps fully integrated in the CDU like the S201 and C051 by ATMOS and the Thopaz and Thopaz+ by Medela. Whatever the method, proper suction must be guaranteed when air leaks are present and this can be achieved only with valves designed for low-pressure high-flow rates [25,26,27]. Powerful hospital central vacuum sources and wall-mounted pressure regulators with large orifices satisfy this requirement, although central vacuum being distributed in a parallel fashion to each room results in a certain loss of vacuum to the wall, especially when many are at work simultaneously. Such loss, anyway, is not relevant if we look at the working ranges (−600 cmH_2_O centrally, −40 cmH_2_O to the CDU). Add-ons and low-flow low-vacuum pumps are newer systems engineered to work at low flow and pressure ranges, hence they have intrinsic limits to the flowrates, they are restrictive to the flow itself and are slow to react. We believe that this, in turn, increases the risk for pneumothorax, subcutaneous emphysema and even more serious events.

It is also important to know exactly how much suction we apply to the pleural space, and how this co-varies with the patient’s breathing. The Sentinel Seal by Covidien is the only CDU offering this feature by means of a fourth chamber. This additional chamber to the conventional three-bottle system is a dedicated patient assessment manometer made by a graduated U-tube filled with water. Water levels provide a direct, continuous reading of the actual intrapleural pressure. This is particularly useful to adjust wall-suction to the truly desired vacuum for each patient. In fact, all other dry-suction CDUs have rotary controls with suction levels indicated as numbers, but such levels do not always match reality [28].

Finally, the dogma of chest drainage systems being “just boxes” without questioning may in fact not be true. Chest drainage started thanks to Gothard Bülau in the late 1800s, and the desire to make the process more compact has led to the design of many other devices in common use [29,30]. Even so, widely different design characteristics led to heterogeneous clinical performances hindering communications between scientists [31,32] and preventing clinicians from providing manufacturers with proper clinical guidance. Inconsistencies in the interpretation of air leaks and intrapleural pressure and in chest tube management are contributors to the conflicting results found in the literature [33]. Altogether this led to phenomena that impact patient care, setting the stage for problems that may not have been present in the past with other CDUs [15,34,35].

Forward-looking colleagues investigated technical aspects of CDUs decades ago already, drawing attention on how certain design details can become clinically relevant. Unfortunately, no substantial traction followed and clinicians continued to accept a status quo laid down by empirical observations passed on from one generation to another.

In 2011, consensus definitions to promote an evidence-based approach to the pleural space were published [36]. On one hand, it was a step forward over prior habits-based chest drainage management. On the other hand, the lack of a comprehensive analysis of the pros and cons of traditional thoracic drainages in favor of characteristics built-in exclusively in electronic systems represented a major source of bias [37]. Indeed, evidence-based medicine (EBM) falls short of making medicine as effective as it can be [38] and, therefore, science-based medicine (SBM) is preferred [39,40,41].

Despite the fact that newer “digital” models are not necessarily superior to traditional ones [42], nor are they needed for every patient, in 2017 the Society for Translational Medicine recommend using electronic (or “digital”) drainage systems for patients undergoing elective lobectomy [43]. In 2019, the European Society of Thoracic Surgeons worked on enhanced recovery after lung surgery, strongly recommending electronic drainage systems, albeit this is still based on low-quality evidence [44]. In 2021, Chopra and colleagues analyzed drainage dependent air leaks, finally linking clinical outcomes to CDU design [45].

As we become busier and busier navigating the administrative burdens in the paperwork crisis, little time is left for us to choose a CDU based on reason, evidence and assessment of prioritized patient needs [6,46]. Furthermore, it has been proven that the interaction with medical representatives can influence the adoption of medical devices by physicians [47,48,49,50]. With regard to CDUs, each of the various companies has claimed non-inferiority or even superiority of their CDUs over competitors. Importantly, most medical personnel have no engineering background to allow rational choice of a CDU [51,52]. Finally, hospital administrators make purchases based on costs, with an overall risk to focus more on price reduction rather than on gaining the insight needed to ensure patients’ health.

There is also a need for personalized post-operative management in thoracic surgery [53,54,55,56,57,58,59,60,61] and we hope that this paper will stimulate discussion on how we are using what is available on the market [62] and on how we need to push for what has still to be developed [63,64].

Better CDUs design requires taking into account the context of use and our clinical perspective as clinicians [65,66]. The design of CDUs interacts indeed with their safety profile and this dipole of intended performance and safety is the basis for capitalizing on future technologies without exposing users and patients to unnecessary risks [67,68,69,70,71]. Thus, the ideal CDU should have the following core features: never to be restrictive to flow, to be quick and to automatically compensate for intrapleural changes, and to integrate real-time digital visual feedback or air leaks and intrapleural pressure (Table 4).

## 5. Conclusions

Chest drainage unit design and performance differ dramatically among devices, and this has implications after thoracic surgery. Changes to CDUs’ mechanism of function may lead to better results, as we demonstrated with a digitally controlled high-flow low-resistance valve providing instant help to a simulated patient with obstructed breathing.

The optimal management of post-lung resection patients is still unclear and we think that this is also due to CDUs not being totally understood. Unfamiliarity with heterogeneous technologies can lead to misinterpretation of the clinical data, hence misdiagnosis, ultimately ending in CDUs failing to support clinical decision making. This also creates a formidable barrier to tailoring chest drainage therapy to each individual patient.

There is a need for better safety and performance requirements and for clinical testing of new designs against traditional ones, as new technology emerges. This may enable personalized medicine concepts applied to chest tube drainage.

## 6. Patents

**Title:** “Device: system and method to customize chest drainage therapy”. **Applications no.** 3,017,252 (Canada) and 261,603 (Israel): granted. **Applications no.** 16/082,091 (USA), 17,763,899.6 (Europe), 2018/010731 (Mexico) and 2018-548094 (Japan): pending.

## Figures and Tables

**Table 1:** CDUs collected for testing and their product design engineering. Pediatric, pneumonectomy and ambulatory units not included.

Manufacturer	Unit Name	Canister Chambers (n)	Water-Seal Chamber Characteristics				Suction Control	CDU Type
			Reservoir Size (mL)	Water Manometer ≥20 cmH_2_O	Delaying Mechanism			
					Float Valve	Notch		
**Atrium/Maquet** **(Getinge Group)** (Gothenburg, Sweden)	Express	3	Optional	-	-	-	Dial	Dry-Dry
	Oasis		45	Yes	Yes	Yes		Wet-Dry
	Ocean						Water level	Wet-Wet
**Bio-Thorametrix** (Gronsveld, The Netherlands)	High capacity	3	60	Yes	No	No	Water level	Wet-Wet
							Dial	Wet-Dry
	Single	1	100	Yes	*-*	*-*	3rd bottle	Wet-Wet
**Cardinal Health/Covidien** **(Argyle line)** (Dublin, OH, USA)	Altitude	3	80	Yes	Yes	No	Dial	Wet-Dry
	Sentinel Seal	4	90					
	Aqua-Seal	3	45				Water level	Wet-Wet
	One Bottle	1	400		*-*	*-*	3rd bottle	Wet-Wet
	Thora-Seal I	1	370		*-*	*-*	2nd CDU	Wet-Wet
	Thora-Seal II	2 (in series)	120		*-*	*-*	Water level	Wet-Wet
	Thora-Seal III	3	110		No	No	Water level	Wet-Wet
**Eurosets** (Medolla, Italy)	Rome	3	45	Yes	Yes	Yes	Dial	Wet-Dry
	Venice						Water level	Wet-Wet
**Meditea/HMC** (Mirandola, Italy)	Thoraflow	1	200	Yes	-	-	3rd bottle	Wet-Wet
**Redax** **(Drentech line)** (Poggio Rusco, Italy)	Chest	1	500	Yes	-	-	2nd CDU	Wet-Wet
	Compact	3	45	No	Yes	No	Water level	Wet-Wet
	Variant dry						Dial	Wet-Dry
	Simple 3 Mobile	2					Add-on PSU	Wet-Dry
	Dune	3	Optional	-	-	-	Dial	Dry-Dry
	Simple Simple Plus	2	70	No	Yes	No	Add-on PSU	Wet-Dry
**Rocket medical** (Washington, UK)	Rocket BLUE	1	500	Yes	-	-	Add-on PSU	Wet-Dry
**Teleflex** **(Pleur-evac line)** (Wayne, PA, USA)	A6000 Cactus	3	70	Yes	Yes	Yes	Dial	Wet-Dry
	A7000						Water level	Wet-Wet
	Sahara 1100		Optional	-	-	-	Dial	Dry-Dry
**ATMOS** (Lenzkirch, Germany)	S201	1	Optional	-	-	-	Electronic pump	Dry-Dry
	S201 re-style							
	C051		No					
**Medela** (Baar, Switzerland)	Thopaz	1	No	-	-	-	Electronic pump	Dry-Dry
	Thopaz+							
**Total**	**32**							**3**

**Table 2:** CDUs classification based on their mechanism of function.

Seal	Suction	Classification
Wet	Wet	
Wet	Dry	Thoracic drainage
Dry	Dry	
		Low-flow/low-vacuum pump

**Table 3:** Vacuum conditions and mechanisms to initiate reverse air flow in UWSD.

Manufacturer		Vacuum Conditions Permitting Reverse Air Flow		Mechanisms		Circumference Notched
		Level (cmH_2_O)	Duration (Seconds)	Emptying Reservoir	Bending Surface	
Atrium/Maquet		25	64	X		Yes
Bio-Thorametrix		28	29	X		Yes
		80	1		X	
Cardinal Health/ Covidien	Aquaseal	30	20	X		No
		35	3		X	
	Thoraseal III	24	18	X		
		40	7		X	
Eurosets		24	42	X		Yes
		63	1		X	
Redax		131	244	X	X	No
		25	1	X		
Teleflex		29	119	X		Yes

**Table 4:** Features to be built-in in the ideal CDU.

To include the functions of the three-bottle system
To integrate a dedicated patient assessment manometer (the “fourth bottle”)
To have automatic positive and negative pressure relief valves
To quickly compensate for pressure changes
To allow high-flow rates at low-pressures
To display real-time data on air leaks and intrapleural pressures for instant clinical use, and to store them for clinical multi-disciplinary discussion and future research purposes
To warn medical personnel of any sudden intrapleural change
To adjust therapy as patients recover
To work in synchrony with mechanical ventilators
To allow direct assessment of ‘true’ or ‘false’ air leakage
To include an automated line-clearing chest tube system
To include an independent source of vacuum
To be portable
To be a ‘home medical device’ with remote control
